# Caffeic Acid Reduces the Viability and Migration Rate of Oral Carcinoma Cells (SCC-25) Exposed to Low Concentrations of Ethanol

**DOI:** 10.3390/ijms151018725

**Published:** 2014-10-17

**Authors:** Arkadiusz Dziedzic, Robert Kubina, Agata Kabała-Dzik, Robert D. Wojtyczka, Tadeusz Morawiec, Rafał J. Bułdak

**Affiliations:** 1 Department of Conservative Dentistry with Endodontics, School of Medicine with the Division of Dentistry, Medical University of Silesia in Katowice, Akademicki 17, 41-902 Bytom, Poland; 2 Department and Institute of Pathology, School of Pharmacy and Division of Laboratory Medicine in Sosnowiec, Medical University of Silesia in Katowice, Ostrogórska 30, 41-200 Sosnowiec, Poland; E-Mails: rkubina@sum.edu.pl (R.K.); adzik@sum.edu.pl (A.K.-D.); 3 Department and Institute of Microbiology and Virology, School of Pharmacy and Division of Laboratory Medicine in Sosnowiec, Medical University of Silesia in Katowice, Jagiellońska 4, 41-200 Sosnowiec, Poland; E-Mail: rwojtyczka@sum.edu.pl; 4 Department of Oral Surgery, School of Medicine with the Division of Dentistry, Medical University of Silesia in Katowice, Akademicki 17, 41-902 Bytom, Poland; E-Mail: tmorawiec@sum.edu.pl; 5 Department of Physiology, School of Medicine with the Division of Dentistry, Medical University of Silesia in Katowice, Jordana 19, 41-808 Zabrze, Poland; E-Mail: rbuldak@sum.edu.pl

**Keywords:** caffeic acid, ethanol, squamous cell line SCC-25, MTT/LDH cytotoxicity assay, cell migration assay

## Abstract

Alcohol increases the risk of carcinoma originated from oral epithelium, but the biological effects of ultra-low doses of ethanol on existing carcinoma cells in combination with natural substances are still unclear. A role for ethanol (EtOH), taken in small amounts as an ingredient of some beverages or mouthwashes to change the growth behavior of established squamous cell carcinoma, has still not been examined sufficiently. We designed an *in vitro* study to determine the effect of caffeic acid (CFA) on viability and migration ability of malignant oral epithelial keratinocytes, exposed to ultra-low concentrations (maximum 100 mmol/L) EtOH. MTT (3-[4,5-dimethylthiazol-2-yl]-2,5-dimethyltetrazolium bromide) and LDH (lactate dehydrogenase) assays were used to assess the cytotoxic effect of EtOH/CFA and the viability of squamous carcinoma SCC-25 cells (ATCC CRL-1628, mobile part of the tongue). Tested EtOH concentrations were: 2.5, 5, 10, 25, 50, and 100 mmol/L, along with an equal CFA concentration of 50 μmol/L. Carcinoma cells’ migration was investigated by monolayer “wound” healing assay. We demonstrated that very low concentrations of EtOH ranging between 2.5 and 10 mmol/L may induce the viability of oral squamous cell carcinoma cells, while the results following addition of CFA reveal an antagonistic effect, attenuating pro-proliferative EtOH activity. The migration rate of oral squamous carcinoma cells can be significantly inhibited by the biological activity of caffeic acid.

## 1. Introduction

Diet of individuals, habits, exposure to environmental chemicals and genetic susceptibility based upon polymorphic variations among genes affect allelic mutations, type and quantity of DNA damage also in oral keratinocytes [1]. It is well proven that excessive ethanol consumption increases the risk and growth of neoplastic lesions of the oral soft tissues, but the biological effects of short exposure of epithelial cells to ultra-low concentrations of ethanol (EtOH) in combination with some natural substances, are still unclear and undiscovered. A role for ethanol, taken in small amounts, e.g., as an ingredient of some beverages or mouthwashes to change the growth behavior of established oral squamous cell carcinoma (OSCC), has been less studied. However, the metabolism of EtOH to create DNA damage through synthesis of acetaldehyde (ALD), a possible carcinogen even at low concentrations, cannot be overlooked because this is a likely occurrence late in the carcinogenesis process. Alcohol metabolism occurs also in the oral cavity and various bacteria in plaque can metabolize alcohol to ALD [1].

According to previous reports, there is an association between the use of domestic mouthwashes containing very low concentration of EtOH and oral cancer [2,3], but data available are inconclusive [4]. McCullough suggested an increased risk from alcohol containing mouthwashes for oral hygiene in the development of oral cancer [2]. Other papers have reviewed the evidence linking alcohol containing mouthwashes as a risk for the development of oral cancer, *i.e.*, the type of head and neck cancerous tissue growth located within the oral cavity and also cancer of the mucosa surfaces of the upper aerodigestive tract [4]. The use of an alcohol-containing mouthwash may lead to short-term formation of ALD in the oral cavity, which is detectable in the saliva for up to 10 min [5]. The concern related to the alcohol content of mouthwashes has led to the development of alcohol free preparations.

According to the literature, natural compounds including phenolic ingredients may impose an anti-tumor activity against malignant lesions, including those located within the oral cavity [6,7]. Caffeic acid (CFA), a naturally occurring phenolic compound, is a component of numerous natural substances including propolis. Although the exact mechanism of the action of the phenolic acids has not been fully established, several potentially important drug-related activities have been reported [8,9,10]. Another form of CFA—caffeic acid phenethyl ester (CAPE)—an active component of propolis, has been implicated in the regulation of cell growth and apoptosis [11,12]. It exhibits antioxidant, anti-inflammatory, antiproliferative, cytostatic, and most notably, antineoplastic properties [13,14].

Propolis used extensively in traditional medicine, seems to be efficient against different tumor cells both *in vitro* and *in vivo*, which suggests its potential in the development of new anticancer drugs [15,16]. It is a semisolid mixture of organic resin and wax, produced by honeybees (*Apis mellifera*), and is used by bees to seal their honeycombs and to protect the entrance against intruders. It is assumed that the chemical composition of propolis comprises approximately 50% of resin and vegetable balm, 30% of wax, 10% of essential and aromatic oils, 5% of pollens, and 5% of other trace substances, including organic debris, depending on the place and time of collection. The constituents of propolis vary widely, depending on the climate, season, location, or year, although its chemical composition is not stable. Anti-tumor effects of propolis extracts and its constituents, e.g., flavonoids, terpenes, galangin and CAPE, have been widely described in the literature because it is composed of a complex mixture of natural substances [17,18,19]. The effect of propolis on experimental carcinogenesis may reveal the possible mechanisms of action against tumors, involving apoptosis, cell cycle arrest and interference on metabolic pathways. Ozturk suggested that CAPE possess anticancer and apoptosis inducing activities [17]. Lee *et al.* showed that caffeic acid-conjugated chitosan proved an anti-proliferative effect against tumor CT26 colorectal carcinoma cells [8]. Xiang *et al.* concluded that CAPE seems to inhibit β-catenin/T-cell factor signaling in colon carcinoma cell lines [11].

Excessive alcohol consumption, particularly in combination with cigarette smoking, increases the risk of OSCC, a common malignant epithelial tumor [20,21]. Epidemiological data deliver evidences of the consistent impact of exogenous carcinogenic factors, such as alcohol drinking, tobacco use and HPV exposure in the development of oral cancers throughout the world [22,23]. Conducted studies indicate the influence of the combination of other etiological factors, such as specific genotype [24], chronic inflammation [25] and/or the synergism with viral and bacterial infection [26]. Moreover, alcohol drinking along with exposure to another factor may increase the risk of oral premalignant lesions in individuals who have never had a smoking habit [27]. Using an animal model, studies indicated that ethanol applied to the oral epithelium produces tissue hyperproliferation. Alcohol on its own causes epithelial atrophy of oral mucosa, and decrease in basal cell size atrophy with associated hyper-regeneration [28]. The dose-dependent effect of ethanol is significant and ingested alcohol from beverages, with their prolonged, repeated exposures, and numerous impurities plays a crucial role in oral carcinogenesis [22].

Despite early diagnostic efforts, advanced radiotherapy and chemotherapy, survival rate of patients with oral carcinoma, carcinoma incidence and mortality have not improved significantly [20,29]. Moreover, a substantial number of patients treated with OSCC may develop a second cancer lesion within a few years. Hence, it is vitally important to understand the etiology and mechanism of OSCC and establish the effective preventive strategies, including novel anti-tumor agents. Epidemiological data have established an etiologic association between exposure to ethyl alcohol (EtOH) and oral mucosa incidence of malignant transformation and OSCC, but there is still a need to further evaluate this relationship with a focus upon established malignant cell types, particularly within the oral environment enriched with extrinsic substances exhibiting biological activity.

Taking into account all the facts above, we made an attempt to assess *in vitro* the effects of very low concentrations of ethanol (EtOH) alone and in combination with biologically active compound CFA on human squamous cell carcinoma cell line (SCC-25) of the mobile part of the tongue. We provided concentration/time-profiles over a limited period of time of 48 h. The results were used for a quantitative assessment of oral carcinoma cells’ viability using the reference MTT and LDH assay. The effect of selected concentrations of EtOH and CFA on oral cancer cell motility and migration was evaluated simultaneously.

## 2. Results and Discussion

**Figure 1:**
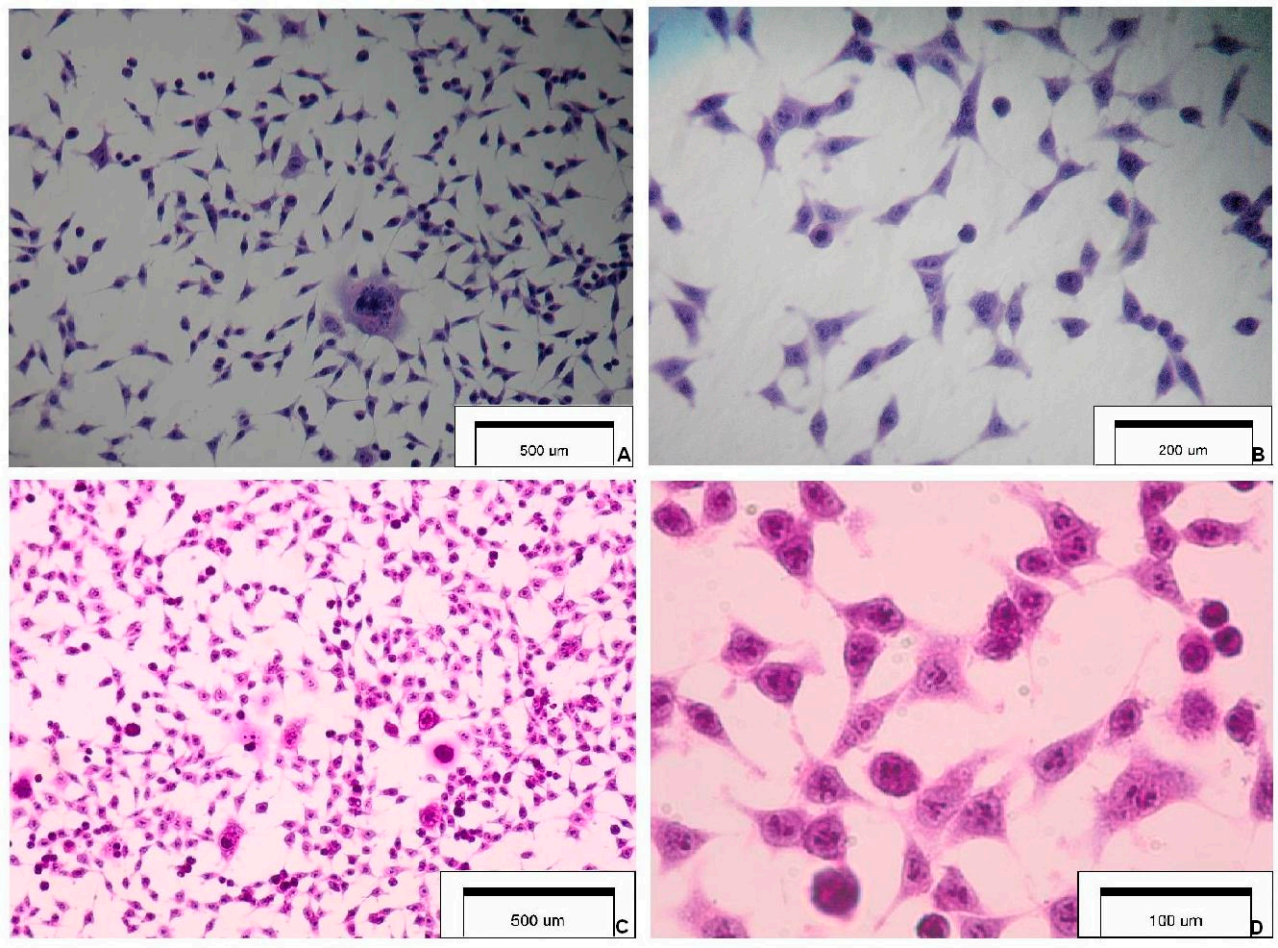
Cytology and morphology of investigated SCC-25 carcinoma cells. Hematoxylin & eosin staining (optical magnification ×100, ×400, ×600). The main cytological features: (**A**) highly irregular shaped cells (tadpole, caudate), irregular nuclear shapes, multinuclear large cell; (**B**) elongated cells, coarse dense chromatin, cells with an increased nucleus:cytoplasm ratio and a malignant-looking nucleus; (**C**) pleomorphism, nuclear enlargement, single giant cells, cells maintaining cohesiveness; and (**D**) cytoplasmic density, chromatin granularity, hyperchromasia, prominent nucleoli.

Our study was designed to research the pro-proliferative effect of EtOH alone and to determine for the first time, whether the addition of active phenolic substance CFA may attenuate the cancer-promoting effect of ethanol. MTT assay used detected succinic dehydrogenase activity in the SCC-25 carcinoma cells mitochondrion and an oxidative metabolic surrogate marker. Figure 1 presents the selected features of SCC-25 carcinoma cells.

**Figure 2:**
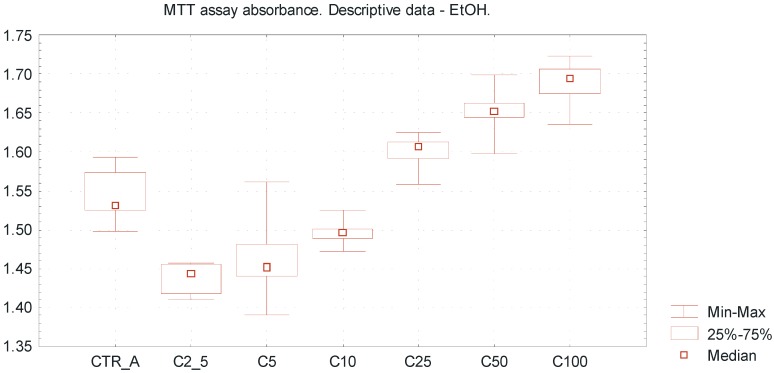
Viability of oral squamous cell carcinoma cells using MTT cytotoxicity assay, exposed to EtOH alone. Descriptive statistics of MTT cytotoxicity assay for EtOH exposure. The “U-shape” trends representing values of MTT absorbance for increasing EtOH concentrations ranged from 2.5 up to 100 mmol/L. Ultra low ethanol concentrations between 2.5 and 10 mmol/L reveal a slight inhibitory effect towards SCC-25 line cells, whereas investigated higher concentrations of ethanol (25–100 mmol/L) promote viability of SCC-25 cells, reflected by pro-proliferative action. *X* axis—EtOH concentrations [C]; CTR_A—control sample A; *Y* axis—absorbance values.

**Figure 3:**
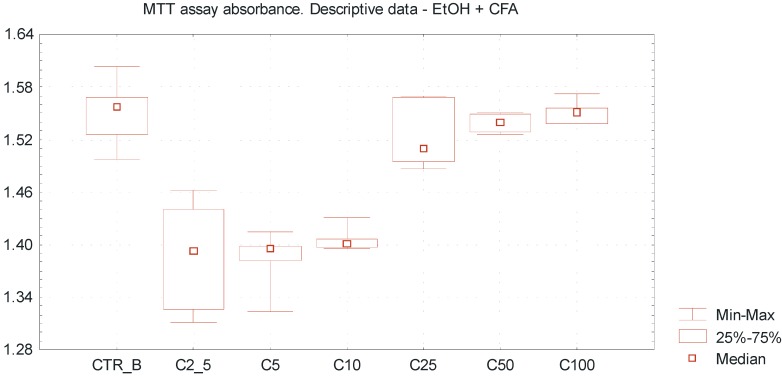
Dose-dependent effect of EtOH alone and combination of EtOH with caffeic acid (CFA) on SCC-25 cells proliferation assessed by MTT absorbance measurement. Higher cytotoxicity of combination of EtOH and CFA (50 μmol/L) compared to the separate action of ethanol alone, accompanied by decreased viability of SCC-25 cells. Statistically significant differences considering the mean absorbance values between group A and group B, comparing the same EtOH concentrations (for the EtOH concentrations above 2.5 mmol/L). A significant variation of MTT absorbance values compared to ethanol alone. *X* axis—EtOH concentrations [C]; CTR_B—control sample B; *Y* axis—absorbance values.

Table 1 represents the descriptive data of obtained results, including absorbance values, percentage of cell viability and tested EtOH concentrations. The overall trends including MTT absorbance value (min–max, median) for different EtOH concentrations are presented in Figure 2 and Figure 3. The concentrations range of ethanol above 25 mmol/L increased the viability of a SCC-25 cells culture to more than 100%; this may be due to a certain extent to reduction of apoptosis compared to non-treated cell cultures (Table 1).

**Table 1:** Descriptive statistics for absorbance measurements. MTT (3-[4,5-dimethylthiazol-2-yl]-2,5-dimethyltetrazolium bromide) cytotoxicity assay [ethanol concentrations units—mmol/L].

Sample	Absorbance [mean]	SD	Max	Min	Median	Cytotoxicity [viability, %]
Ethanol [Group A]						
Control A	1.542	0.035	1.593	1.498	1.531	100.00
EtOH 2.5 mmol/L	1.438	0.021	1.457	1.411	1.444	93.27
EtOH 5 mmol/L	1.463	0.057	1.561	1.390	1.452	94.86
EtOH 10 mmol/L	1.497	0.018	1.525	1.472	1.497	97.06
EtOH 25 mmol/L	1.600	0.023	1.625	1.559	1.607	103.78
EtOH 50 mmol/L	1.651	0.033	1.699	1.597	1.653	107.09
EtOH 100 mmol/L	1.687	0.031	1.723	1.635	1.694	109.45
Ethanol + Caffeic Acid (CFA) [Group B]						
Control B	1.540	0.034	1.604	1.498	1.539	100.00
EtOH 2.5 mmol/L + CFA	1.388	0.063	1.462	1.312	1.393	90.11
EtOH 5 mmol/L + CFA	1.385 *	0.032	1.415	1.323	1.396	89.92
EtOH 10 mmol/L + CFA	1.405 *	0.013	1.431	1.396	1.401	91.24
EtOH 25 mmol/L + CFA	1.523 *	0.037	1.569	1.486	1.510	98.89
EtOH 50 mmol/L + CFA	1.539 *	0.010	1.551	1.526	1.540	99.93
EtOH 100 mmol/L + CFA	1.551 *	0.013	1.572	1.538	1.551	100.71

Control A, sample without EtOH; Control B, sample with CFA; *, statistical sagnificance at *p* < 0.05.

Very low EtOH concentrations between 2.5 and 10 mmol/L caused a slight inhibitory effect towards SCC-25 cells proliferation, whereas higher concentrations of EtOH (25–100 mmol/L) promoted viability of the investigated cells, reflecting by pro-proliferative action (Figure 2). Differences between the mean absorbance values within group A were statistically significant (ANOVA Fiedmann test, *p* < 0.005). It was observed that caffeic acid enhances the cytotoxic effect of ethanol above a concentration of 25 mmol/L, reflected by the increase of MTT assay absorbance value and inhibition of SCC-25 cells proliferation (Figure 3). Differences between the mean absorbance values within group B were statistically significant (ANOVA Fiedmann test, *p* < 0.005). The overall cytotoxicity of the combination of EtOH in low concentrations and CFA (equal amount 50 μmol/L in each sample) was higher than the separate action of EtOH alone, accompanied by decreased number of vital SCC-25 cells. The differences of mean absorbance values between group A and group B, considering the same EtOH concentrations, were statistically significant for the EtOH concentrations above 2.5 mmol/L (Wilcoxon test; *p* < 0.05).

**Figure 4:**
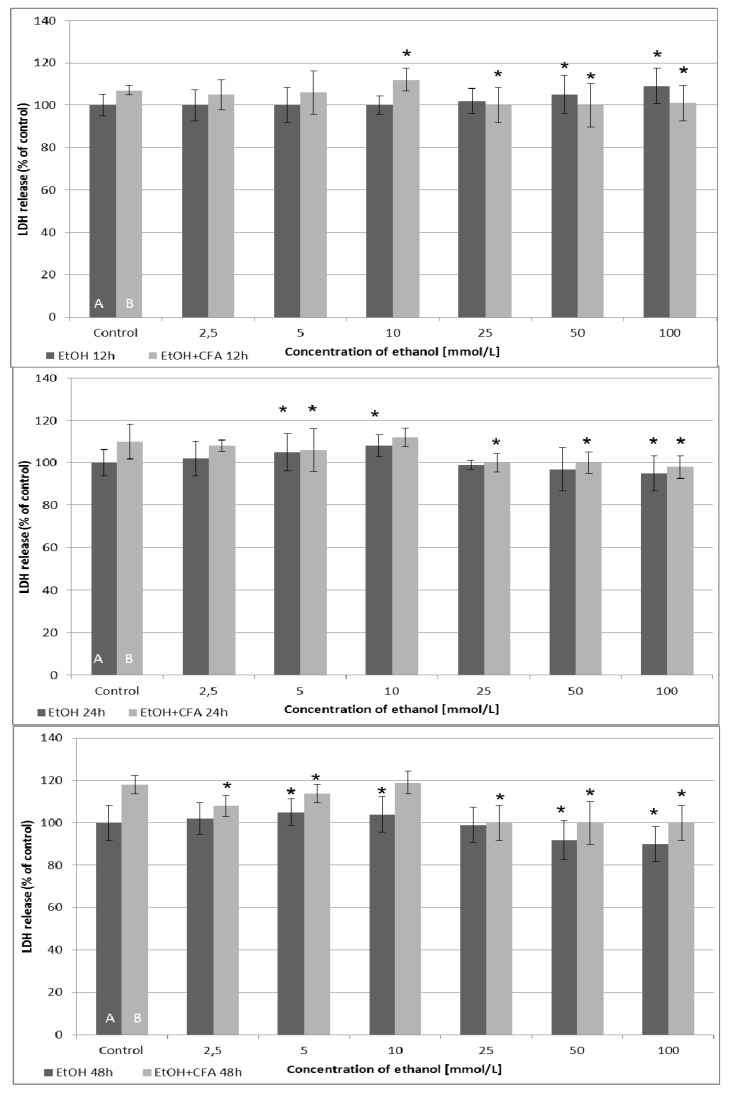
Cytotoxic effects measured by lactate dehydrogenase (LDH) enzymatic activity in human tongue carcinoma cells and after treatment with CFA and EtOH. LDH levels are measured following treatment with 50 µmol/L of CFA for 12, 24 and 48 h (2 and 6 h exposure time is omitted). Ethanol concentration—dependent quantification of LDH activity released from damaged SCC-25 cells. Control A includes native cells and medium alone whereas control B includes native cells, medium and CFA (50 μmol/L) without EtOH. (*) indicates statistically significant differences compared to the control.

Analyzing the results of LDH cytotoxicity assay, the cytotoxic effect was dependent on the time of the EtOH and CFA exposition (Figure 4). After 12 and 24 h of incubation, LDH release was generally higher for mixtures of EtOH and CFA, up to an EtOH concentration of 10 mmol/L. The highest LDH release values were detected for combination of CFA + EtOH 10 mmol/L after 48 h and for CFA alone (positive control) after 48 h. The release of LDH from cells into the culture medium increased after 6 h in EtOH-treated cancer cells compared with untreated cells, for the EtOH concentrations above 10 mmol/L. This suggested that EtOH 25/50/100 mmol/L and CFA/EtOH 100 mmol/L treatment of SCC-25 caused plasma membrane rupture and cellular damage in carcinoma cells after 6 h. Interestingly, the combined CFA + EtOH treatment did not induce significant LDH releases after 24 h compared to 12 h exposure and was less toxic for tested cell lines after 48 h. Our results indicate that after 24 h EtOH has a dose-dependent toxic effect on SCC-25 cells up to a concentration of 10 mmol/L.

**Figure 5:**
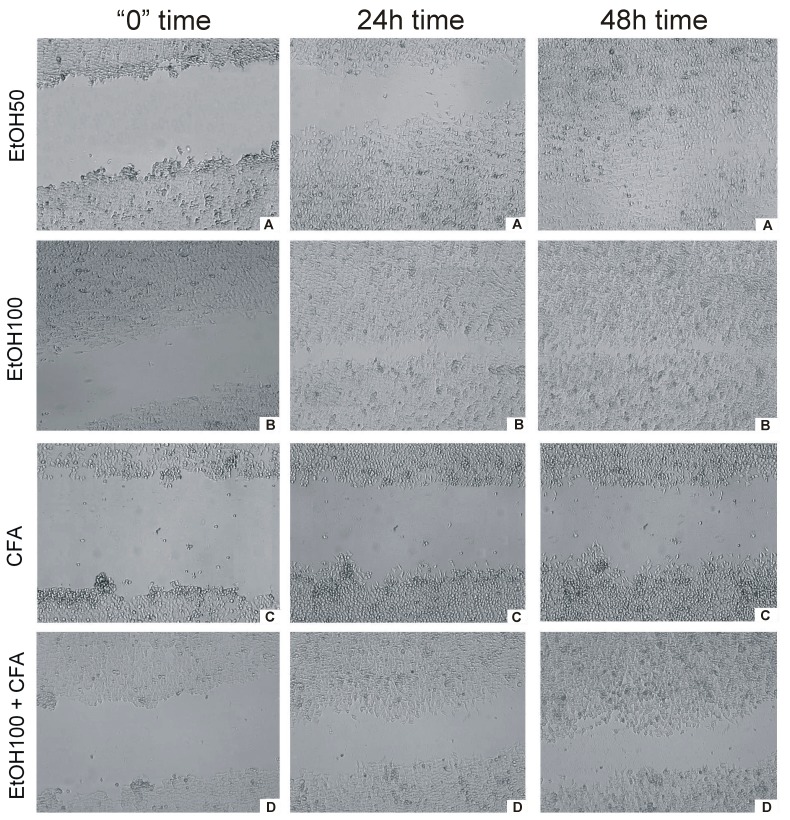
Time-dependent effect of CFA, EtOH and combination of CFA + EtOH on oral carcinoma cells’ migration representing by “wound” closure assay. Images of “wound” closure and migration rate of SCC-25 cells at different time points taken with JuLi FL. (**A**) effect of CFA at three different time points; (**B**) effect of EtOH + CFA at three different time points; (**C**) effect of EtOH50 + CFA at three different time points; (**D**) effect of EtO100 + CFA at three different time points. (**Left** column) cells at start of experiment; (**middle** column) cells after 24 h of experiment; (**right** column) cells at the end of “healing wound” experiment 48 h.

Live cell imaging provided a fast and efficient way to measure the rate of migration of cells into a wound space without the need for operator intervention. In the wound-healing assay, CFA alone was clearly shown to inhibit cell migration represented by the lowest rate of wound closure. OSCC cells migrated more slowly and CFA did affect the migration rate of SCC-25 markedly and time dependently. EtOH-treated cancer cells covered *ca.* 100% gap at 30 h, *i.e.*, almost the entire scratched area. In contrast, the scratch was still uncovered at 48 h in CFA-treated cancer cells and CFA/EtOH-treated cells (Figure 5). Interestingly, the mixture of CFA/EtOH inhibited migration of SCC-25 cells, but substantially less than CFA alone.

**Figure 6:**
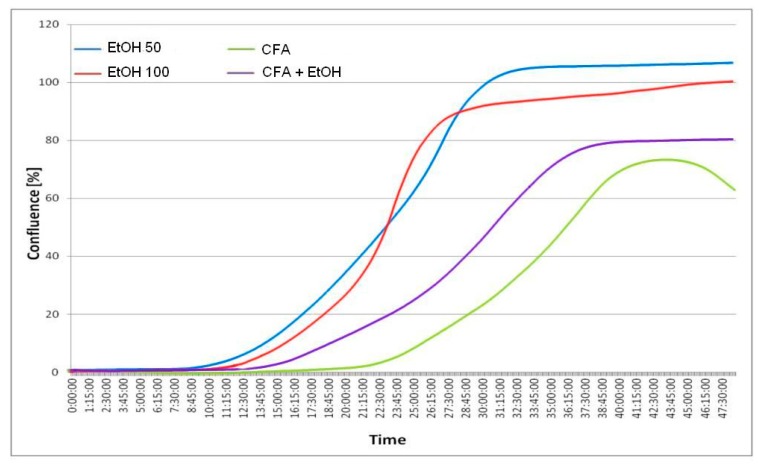
Time *vs.* % confluence function curves (graph below). Only about 5% wound closure is visible after 12 h for EtOH and lack of closure for CFA agent and CFA/EtOH mixture. Complete or almost complete closure occurs after 30 h for EtOH 50 mmol/L and EtOH 100 mmol/L, while CFA (50 μmol/L) significantly effects migration of cancer cells, with only 20%–40% of wound closure after 30 h. The least closure (below 80%) is observed for pure CFA (50 μmol/L) agent after 48 h. The selected images are representative of the experiment duration.

These results indicate that CFA suppresses the motility of SCC-25 cells more than in combination with ethanol 100 mmol/L. Cellular migration was altered by either 50 and 100 mmol/L of EtOH. The motility suppression was associated with reduced viability of the tested cells. These findings were observed for CFA single concentration—50 μmol/L. Our results indicated increased inhibition of cell migration with increasing EtOH concentration. When treated with EtOH, the migration of squamous cell carcinoma cells appears to be less affected at low concentration (50 mmol/L) but at the highest concentration (100 mmol/L EtOH) the migration of cancer cells was significantly inhibited (Figure 5 and Figure 6).

Despite the fact that numerous epidemiological studies have confirmed excessive alcohol drinking as a major risk factor for human oral cancer, a relationship between EtOH doses (concentrations) and epithelial cells neoplastic progression has not been developed to clearly describe ethanol-related oral carcinogenesis [22,23,24,30]. In our *in vitro* study ethanol and antitumor agent were combined together to understand the concentration-varying effect of ethanol on the carcinoma cells proliferation. The mechanism of ethanol-related carcinogenesis, *i.e.*, how ethanol may promote the neoplastic oral keratinocytes is still unclear. *In vitro* studies have demonstrated that alcohol enhances the mucosal penetration of the various carcinogens found in tobacco [31]. It is known that ALD reveals a mutagenic effect and animal studies have shown this substance to be carcinogenic [1,28]. What is more, ethanol may interfere with arachidonic acid metabolism at multiple levels [32].

We observed a bidirectional “stimulative-inhibitory phenomenon” considering the investigated ultra-low concentrations of ethanol. The ultra-low concentrations of ethanol up to 10 mmol/L may induce proliferation of oral sqamous cell carcinoma cells in *in vitro* model, while the simultaneous exposure to ethanol along with CFA resulted in a decreased number of SCC-25 cells. Ethanol concentrations higher than 10 mmol/L seemed to influence the SCC-25 cells revealing a less cytotoxic effect, which resulted in viability values of more than 100%. On the other hand, the exposure of SCC-25 cells to both investigated substances at the same time demonstrated less stimulative effect than ethanol alone, accompanied by a decreased number of SCC-25 cells in *in vitro* conditions. Overall, anti-proliferative and anti-migrative effects of CFA towards the cancer cells determined by LDH assay were highly correlated with the results obtained by the MTT method. Our results demonstrate that the biologically active phenolic compound CFA has a cytotoxic effect on the tested oral carcinoma cell line. CFA was found to be the active principle exhibiting strong inhibition of xanthine oxidase which is related to several diseases, e.g., tumors [33].

Our results could indicate that CFA targets and lethally harms carcinoma cells. It is able to reduce the carcinoma cell viability, disrupting the LDH activity in carcinoma cells and significantly affect carcinoma cell migration. It is assumed that simultaneous CFA and EtOH initial incubation decreased the metabolism and generation of SCC-25 cells DNA damage. The latter effect further assumes additional DNA damage occurred in the form of the synthesis of ALD, with bulky adduct formation from EtOH exposure and also due to CFA exposure and its anti-proliferative activity towards SCC-25 cells. However, the carcinoma cells’ matabolism seemed to be increasing. In conclusion, the overall exposure to both ethanol and CFA produced damage that was repaired sufficiently to permit maintenance of a viable malignant cell state and growth.

The result of this study showed that CFA significantly inhibited the migratory/invasive ability of SCC-25 carcinoma cells. The mechanisms for the regulation of carcinoma cell motility and invasion remain unclear. Results obtained by Chih-Yu Peng *et al.* [13] showed that CAPE attenuated SCC-9 cell migration and invasion at noncytotoxic concentrations (0–40 μM). Chiao-Wen Lin *et al.* observed that kaempferol—which is a flavonol and has a similar structure to quercetin and myricetin—substantially inhibited the migration and invasion of SCC-4 cells in a dose-dependent manner, with only 58% and 56% remaining after a treatment of 100 µM of kaempferol at 24 and 48 h, respectively [34].

Obtained data were partially the same as results from other studies. Castaneda observed that ultra-low ethanol concentrations (equivalent to 1 mmol) inhibit cell proliferation and increase apoptosis more strongly in liver carcinoma cells (HepG2) than in normal hepatocytes [35]. A time-dependent effect of ethanol at low concentrations up to 250 mmol/L on SCC-15 and SCC-4 cell growth as determined by MTT assay, was demonstrated by Guo and co-authors using an *in vitro* study [32]. The upper limit of ethanol concentration at 500 mmol/L was toxic to all cells. They concluded that ethanol promotes 4-nitroquinoline-1-oxide (4NQO) induced oral carcinogenesis through further activation of the 5-lipoxygenase (5-Lox) pathway of arachidonic acid metabolism. Also, ethanol upregulated 5-Lox expression in human tongue SCC cells. Contrary to our results, all concentrations of ethanol revealed an anti-proliferative effect towards tested cells.

Our data were also indirectly in agreement with a recent study conducted by Hager *et al.*, who reported that ethanol induces proliferation of SCC-25 accompanied by an increased number of carcinoma cells in the S phase of the cell cycle [36]. Interesting results obtained by Schwartz *et al.* suggest that oral *Streptococci* spp. and human papilloma virus (HPV-16) cooperate to transform oral keratinocytes after low-level EtOH (1%) exposure [26]. Considering the combine influence of ethanol and phenolic acids on cell methabolism, Lee *et al.* stated that quercetin, catechin, CFA and phytic acid have protective effects against ethanol metabolism-induced oxidative stress in SK-Hep-1 cells by elevating antioxidant potentials [37]. Similar observations towards human HT-1080 fibrosarcoma cell line were revealed by Rejendra *et al.* [38].

It should be stated that natural tongue epithelial cells may respond differently to ethanol exposure compared to squamous cell carcinoma. It is also important to dissociate cell participants as to their role in carcinogenesis and growth behavior of OSCC. Cells in the interior of an OSCC will grow and respond to ethanol or other active substances differently than cells at the tumor growth boundary interface. Ultimately the viability of the mucosa and its integrity affects the exposure of stem cell like keratinocyte populations to extrinsic chemicals and to varying concentrations of these chemicals on the surface in conjunction with saliva quality and quantity.

It needs to be mentioned that saliva, due to the presence of oral bacteria and biofilm interaction with oral mucosa keratinocytes, can modify concentrations of chemicals commonly taken. A microbe biofilm contains individual species, for example *Streptococci* spp., that are capable of metabolizing the ethanol [27,28]. Furthermore, there are additional important metabolic considerations (e.g., free radicals, NO) and other behavior variables that may modify this relationship and ultimately the response of either primary transforming and/or fully malignant human keratinocytes.

Therefore, advanced experiments with immortalized oral epithelial cells would be beneficial to establish the association between SCC cell proliferation and the pro-proliferative effect of ethanol, particularly at the early stage of oral carcinogenesis. Also, further studies are required regarding the anticancer activities of CFA and/or CAPE in medicine in dentistry in order to reveal the clinical potential of CFA, if it is going to be used as an anticancer agent. These studies, including the model of ethanol and bioactive agents revealing a protective activity towards oral soft tissues, may deliver novel findings, which may provide insights on development or suppression of human oral cancer.

## 3. Experimental Section

### 3.1. Tongue Carcinomar Cell Culture

Cell line of human tongue (mobile part) squamous carcinoma SCC-25 (catalogue no. ATCC CRL-1628, American Type Culture Collection, Manassas, VA, USA) was cultured with the use of a mixture of a 50:50 proportion of Dulbecco’s modified Eagle’s medium (DMEM) and Ham’s F12 medium with an addition of 10% inactivated fetal bovine serum—FBS. Medium was supplemented with 400 ng/mL hydrocortisone and Antibiotic–Antimycotic Solution (final concentration: 100 U/mL penicillin, 100 μg/mL streptomycin and 0.25 μg/mL amphotericin B). The carcinoma cells were maintained and grown in monolayer cultures at 37 °C and 5% of carbon dioxide (CO_2_) incubator, Heraeus Instruments, Hanau, Germany). Subsequent passages were made by treating confluent cell culture with trypsin solution and then cells were plated into a new cell culture receptacle in the ratio of 1:4. All reagents for cell culture were purchased from PAA Laboratories GmbH (Pasching, Austria) and Sigma–Aldrich Chemical Company (St. Louis, MO, USA).

### 3.2. Hematoxylin and Eosin Staining Protocol

After fixation in 96% ethanol for 12 h, the SCC-25 cells were rehydrated with a graded series of decreasing concentrations of ethanol (99.6%; 96%; 90%; 80%; 70%; 50%), stained for 12 min with hematoxylin, washed with phosphate-buffered saline (PBS) for development of the blue color for 30 min, and then incubated with eosin for another 30 s. During the next stage, the cells were washed with PBS and dehydrated using a graded series of increasing concentrations of ethanol (50%; 70%; 80%; 90%; 96%; 99.6%). The slides were left in a solution of ethanol:xylene (50:50) in the tank for at last 1 min and then they were exposed to pure xylene for 1 min. The mounted slides were visually assessing with a Zeiss Axiostar binocular microscope (Carl Zeiss, Jena, Germany). All reagents were purchased from Avantor Performance Materials (Gliwice, Poland). Figure 1 presents selected images of investigated SCC-25 cells.

### 3.3. Cell Viability

As an indicator of cytotoxic activity of ethanol and caffeic acid an MTT (3-[4,5-dimethylthiazol-2-yl]-2,5-dimethyltetrazolium bromide) assay was used. It is a colorimetric method consisting of the evaluation of neoplastic cells’ metabolic activity, using the conversion of tetrazolium salts in mitochondria into an insoluble formazan product. The amount of created formazan is proportional to the amount of living cells. MTT assay detects succinic dehydrogenase activity in the mitochondrion and is an oxidative metabolic surrogate marker. In order to evaluate EtOH and CFA cytotoxic properties, cells of examined lines were plated on 96-well plates in the amount of 10,000 cells per well and then culture medium in an amount of 0.1 mL was added. Cancer cells were left for 24 h in order to attach to the culture medium. After lapse of this period, culture medium was decanted and to each well a culture medium containing ethanol (Avantor Performance Materials, Gliwice, Poland) with concentrations of 0, 2.5, 5, 10, 25, 50, and 100 mmol/L was added and left for 48 h. Additionally, we used culture medium containing ethanol and the same concentration 50 μmol/L of caffeic acid—CFA (Sigma–Aldrich, St. Louis, MO, USA). After the lapse of this period, to each well 10 μL of MTT reagent (Sigma–Aldrich) was added. Cells were left for 4 h. Culture medium was decanted and to each well 200 μL of dimethyl sulfoxide (DMSO, Sigma–Aldrich) was added, in order to dissolve the formed formazan crystals. The spectrometric absorbance at 570 nm was measured using a microplate reader (Elx800; Bio-Tek Instruments Inc., Winooski, VT, USA). The percentage of cytotoxicity was calculated by the following formula: percent cytotoxicity (dead cells) = (1 − [absorbance of examined sample/control absorbance]) × 100%.

### 3.4. Lactate Dehydrogenase Release Assay

Additionally, alcohol-induced and CFA-induced cytotoxicity was measured using the LDH Cytotoxicity Detection Kit purchased from Roche (Indianapolis, IN, USA). The standard protocol assays reported here were performed according to the manufacturer’s instructions. This colorimetric assay was used to quantitatively measure lactate dehydrogenase (LDH) released into the media from damaged cells as a biomarker for cellular cytotoxicity and cytolysis. The SCC-25 cells were plated in a 96-well plate (10,000 cells per well) in culture medium supplemented with 10% fetal bovine serum and incubated overnight at 37 °C in 5% CO_2_. The cells were then treated with different concentrations of EtOH (2.5, 5, 10, 25, 50, and 100 mmol/L) and CFA (50 μmol/L) for 24 h before being subjected to the indicated assays. LDH activity was determined by spectrophotometric absorbance using a standard plate reader with a reference wavelength of 490 nm.

### 3.5. Carcinoma Cells Migration Assay

Variable biological mechanisms are involved in carcinoma cell migration. Therefore, we next examined the ability of CFA to modify cell motility using the scratch “wound” healing assay. Oral cancer SCC-25 cells migration was assessed by monolayer “wound” healing (cell scratch) assay, implemented in order to estimate the migration activity and proliferation rates of cells exposed to ethanol and caffeic acid. SCC-25 cells (4 × 10^6^ cells/well) were plated in 6-well plates for 48 h to confluence about 80%, wounded by scratching with a p200 pipette tip. The debris was removed and the edges of the scratch were smoothed by washing the cells once with 1 mL of the growth medium. Scratches were of approximately similar size in the assessed cells and control cells to minimize any possible variation caused by the difference in the width of the scratch.

Then cells were incubated with DMEM medium containing 0.5% FBS, and treated with CFA (50 μmol/L), EtOH (50 and 100 mmol/L) and mixture of CFA + EtOH for 24 h. The negative control sample contained the cells and standard medium without any active ingredients (without EtOH and CFA). Wounds generated healed for 48 h, were processed as above, and wound regions were measured using fluorescence live cell movie analyzer (JuLi FL, NanoEntek, Seoul, Korea) for automate cell confluence analysis and a real-time cell motility measurement. The exposure parameters were as follow: brightness 8%, zoom level 1×, total time 48 h, image capture interval 3 min. Wound confluence was recorded digitally and graphed to quantitatively analyze the recovering of the surface of wound closing with a JuLi FL automatic analyzer.

### 3.6. Statistical Analysis

The results are expressed as mean ± SD obtained from three independent experiments performed in duplicate (*n* = 6). Differences within EtOH alone group A samples and EtOH + CFA samples (group B) were tested for significance using the Friedman ANOVA test (absorbance means values, different EtOH concentrations). Differences between EtOH alone samples (group A) and EtOH + CFA samples (group B) were tested for significance using subsequently the Wilcoxon test (absorbance means values, the same EtOH concentrations; (Statistica 9.0v, StatSoft, Tulsa, OK, USA). A *p* value <0.05 was considered significant.

## 4. Conclusions

The ability of selected phenolic compounds including caffeic acid to augment cancers cells death/apoptosis supports their potential use in chemoprevention strategies for squamous cell carcinoma control, in which malignancy can be prevented or reversed by nutritional or pharmacologic intervention using natural substances. Inhibition of oral carcinoma cell migration by caffeic acid seems to be a beneficial effect of nontoxic phenolic compounds which reveal antitumor activity against neoplastic cells of squamous cell carcinoma. However their pleiotropic effects and mechanism of action are not yet completely understood.
